# Pain and psychiatry: a critical analysis and pharmacological review

**DOI:** 10.1186/1745-0179-2-31

**Published:** 2006-11-06

**Authors:** Donatella Marazziti, Francesco Mungai, Laura Vivarelli, Silvio Presta, Bernardo Dell'Osso

**Affiliations:** 1 Dipartimento di Psichiatria, Neurobiologia, Farmacologia e Biotecnologie, University of Pisa, Italy; 2 Compulsive, Impulsive and Anxiety Disorders Program, Department of Psychiatry, Mount Sinai School of Medicine, New York, NY, USA; 3 Department of Psychiatry, Institute of Biomedical Sciences, Hospital "L. Sacco", University of Milan, Via G.B. Grassi 74, 20157, Milan, Italy

## Abstract

Pain is one of the most difficult medical problems to diagnose and treat and can be a common symptom of several psychiatric disorders. Pain-related issues are heterogeneous and often underestimated or misinterpreted, with the result that psychiatric interventions, which might have been beneficial from the outset, are often delayed or requested only as a last measure. Several problems arise from the definition, classification and assessment of pain, when documented according to the different scales which are commonly used, since these attempt to cover a multitude of analytical requirements, without really succeeding. An area of constant debate regards the connection between pain and various psychiatric disorders, and the difficulty in the classification of pain disorders within the currently existing framework. The pharmacological treatment of pain is complex and implies a variety of different compounds, from opioids to psychotropic medications like antidepressants and anticonvulsivants.

This paper explores the mutual and reciprocal influence between pain and psychiatric disorders reviewing the latest developments in the definition, assessment and treatment of pain, with special emphasis on the impact of pain on psychiatric disorders (and vice versa), and on the use of psychotropic drugs in the treatment of pain syndromes.

## Background

Pain: definition, epidemiology and classification

Pain is a complex experience which includes affective, cognitive and behavioural features, all of which are the result of mental processes and, as such, it represents a psychological condition [1]. The phenomenon of pain, therefore, involves pathophysiological and psychological components that are frequently difficult to interpret. Suffering is a term frequently used in conjunction with pain, implying the conscious endurance of pain or distress and refering to a wide range of intense and unpleasant subjective states that may be of physical or psychologic origin.

The most comprehensive and exhaustive definition of pain is the one provided by the International Association for the Study of Pain, namely "an unpleasant sensation and an emotional experience associated with a real or potential damage to tissue, or the equivalent of such damage"[2]. This definition attempts to overcome the dualism between pain provoked by an evident organic disease and that related to psychological factors, that is, between "real" and "imagined" pain, since clearly in either case, the patient's suffering is real.

Pain is a common problem in the general population and is one of the most frequent factors leading patients to consult a physician [3,4]. When patients suffer from chronic pain – defined as daily pain which has persisted beyond a month or beyond what would normally be considered the appropriate time for recovery from the underlying pathology in question – various specialists are involved in their treatment, but they rarely include a psychiatrist [5-11]. The U.S. Centre for Health Statistics carried out an 8-year follow-up study, which showed that 32.8% of the U.S. civilian population suffered from symptoms related to chronic pain [12]. A recent study carried out by the World Health Organization, which involved more than 25.000 patients in 14 different countries, reported that the 22% of primary care patients had suffered from pain which had been present for most of the time throughout a period of at least 6 months [13]. A review of elderly populations [14,15] shows that the percentage of individuals affected by some kind of pain rises to 50%, and that this is associated with a significant impairment of social functioning and quality of life; furthermore, about one third of chronic pain cases in elderly people are not recognized by their caregivers [16-19].

In spite of its relevance, pain has long been neglected by the past editions of the Diagnostic and Statistical Manual of Mental Disorders (DSM).

Pain "determined" by emotional factors had been classified within the DSM-II amongst psychophysiological disorders [20]. The DSM-III coped with the problem of chronic pain while introducing the concept of psychogenic pain disorder, where the pathophysiological aspects were absent or insufficient to explain the length and the severity of pain [21]. For somatoform pain disorder (DSM-III), psychological factors were no longer required in pain etiology [22].

In the revision of the third edition of the DSM-III (DSM-IIIR), specifications requiring psychological factors to be present, and that the pain should not be due to another mental disorder, were deleted and, instead of considering pain itself, the presence of "pain related problems of at least 6 months' duration" was introduced as the main criterion. In line with these changes, the disorder was defined as "somatoform pain disorder". The literature available indicates that the categories of disorders characterized by pain in DSM-III and DSM-IIIR were rarely used for the diagnosis of patients affected by pain [23]. The evident limitations of the diagnosis of psychogenic and somatoform pain led to the introduction of a new diagnostic group more widely defined in the DSM-IV as "pain disorder".

The DSM-IV introduced this category to better define the diagnostic subgroup of somatoform disorders present in the DSM-III-R, which includes also conversion disorder, hysteria and body dysmorphic disorder, all characterized by the common feature of the presence of a physical symptom, which is suggestive of a medical disorder but has not been provoked by it or by substance use [24,25]. Primary criteria for the diagnosis of pain disorder include 1) pain as the main problem and 2) pain determining significant stress or functional impairment. This diagnosis requires also that although psychological factors may play an important role, the pain must not be the result of another mental disorder, such as an anxiety disorder or depression. Thus, if the presence of another mental disorder is determined, then a diagnosis of pain disorder is not acceptable. Furthermore, it is required that the pain being suffered be defined as acute or chronic and whether associated only with psychological factors or with both psychological factors and a general medical condition. Therefore, the concept of psychological and physical dualism is innate within such a diagnosis.

By definition, a specific medical cause underlying the various somatoform disorders is rarely present [26]; however, in parallel with the increase in "psychogenic" symptoms, functional or social impairments may arise, there may be an increase in medical treatments, inadequate therapeutic strategies or diagnostic examinations, and psychiatric disorders may appear [26,27]. These patients have a higher likelihood of reporting catastrophic thoughts and they tend to think that the origin of their pain is a mystery; they feel that they have lost control and that their physicians do not believe their pain to be real. Patients suffering from chronic pain and presenting symptoms with no medical explanation, suffer also from the iatrogenic consequences of their symptoms, such as an excessive number of medical evaluations and long-term treatments with inadequate drugs [28]. Although the diagnosis of somatization disorder is rare in patients with chronic pain, somatization is a common occurrence in medical practice and it may arise with different degrees of severity and be seen in several forms [29]. Somatization may be conceptualized by three distinct, albeit overlapping, models of behavior, according to which "symptoms may be perceived, evaluated and managed differently by different kinds of persons". These three forms include 1) a high level of symptoms which cannot be explained by physiological systems; 2) somatic pathology, related to worries, which is more severe than would normally be expected under the circumstances (as far as can be determined), and 3) the predominantly or exclusively somatic presentation of symptoms of psychiatric disorders, such as depression and anxiety [30-32]. Environmental factors such as the social stigma of psychiatric disorders, a history of physical or sexual abuse and the medical attention given to biochemical paradigms, predispose and reinforce somatization [33-35].

Other classification systems have been proposed, but none is widely applied: the most comprehensive is that developed by the taxonomy sub-committee of the International Association for the Study of Pain [2] which has set up a model based on 5 axes. Psychological factors may be indicated on both the second axis, where psychiatric disorders may be codified within the central nervous system, and on the fifth axis, where psychophysiological or psychological etiologies may be included.

More controversial, however, is the nosological position of chronic pain syndrome, which is not included anywhere, although this term is frequently used for those individuals suffering from pain for long periods. Black's definition [36], the most widely accepted, applies the following criteria: untreatable and often multiple areas of pain, usually unrelated to any existing somatic problems, previous contacts with various physicians and diagnostic procedures, excessive worry related to the pain and altered behavior including depressive, anxious or neurotic features. However, the validity of this diagnosis is limited because some of the above-mentioned criteria are highly subjective and repeated diagnostic tests might have been due to low awareness of the pain on the part of the physician. For these reasons, the diagnosis of chronic pain syndrome, according to some authors, should be avoided [23].

## Pathophysiology of pain

Different theories have been proposed in this field and, although none is considered exhaustive, the most accepted is the gate control hypothesis [37]. It postulates that the transmission of nerve impulses from the periphery through the central nervous system (CNS) is modulated by T-cells in the substantia gelatinosa of the dorsal horn of the spinal cord, which are influenced by different inputs. These include large inhibitory and small facilitating fibres from the brain, the level of arousal via the reticular activating system and autonomic modulation via the sympathetic ganglia. This theory has considerable heuristic merit and provides a rationale by which emotions or experience could affect pain. A variety of biochemical mechanisms also modulate pain. Analgesia is enhanced by increased levels of serotonin in the periacqueductal grey matter or by potentiating inhibitory substance P in the spinal cord; the opposite may result from the blockade of dopamine or increase of norepinephrine in the lateral reticular formation. These mechanisms indicate the potential effects of biogenic amines and psychotropic drugs on pain, which probably have a modulating but less specific function than brain opiate receptors and the endogenous or exogenous substances acting at their level in both the limbic system and the substantia gelatinosa [38].

## Assessment of pain

It is sometimes difficult to evaluate pain, especially in patients with a terminal disease, with cognitive impairments or other chronic degenerative neurological disorders [9,10]. Different pain rating scales are available to assess the severity and the intensity of pain, but several factors may influence these evaluations, in particular the somatic conditions, the co-occurrence of psychiatric disorders, the presence of stress, the personality characteristics and an interpretation based on subjective experience.

A complete evaluation should be carried out with every patient suffering from pain or agitation or with an evident worsening of his/her functional status. This evaluation should take into consideration simple standardized items to measure the severity of pain, the observation of any pain-associated behavior such as difficulty with daily life activities as a result of pain and a clinical examination in order to localize the pain and any signs which might suggest possible etiological agents.

Given that pain is a subjective experience, several methods have been proposed for its evaluation and measurement [39,40], although none has yet proven universally valid.

The simplest measurement of pain is the *Numeral Evaluation Scale*, where the patient is asked to assign a number to the pain suffered. The typical scale has a range of 0 to 10, with "0" representing a total absence of pain and "10" the worst imaginable pain. Strongly related to this is the visual analogical scale that requires the subject to indicate a point along a line of 10 cm, where 0 is no pain and 10 is the worst [41].

The *McGill Pain Questionnaire *offers a more accurate analysis [42], with the test listing 20 series of words describing the pain and assigned to sensorial, affective and rating scales. The test score may be defined on the basis of the total number of words chosen or by the order in which they are expressed. Some studies, however, indicate that the modality of response varies according to the kind of pain [43]; the McGill Pain Questionnaire has been criticized because it is based excessively on linguistic abilities, and consequently, its results may be influenced by the degree of individual intelligence or education.

An alternative test commonly used is the *West Heaven-Yale Multidimentional Inventory*, composed of 52 items which measure the way in which patients perceive other people's reaction to their pain, participation in everyday life and the effect that the pain is having on their global lifestyle [44].

Other more traditional psychological evaluation tests have also been used, such as the *Minnesota Multiphasic Personality Inventory *(MMPI); nevertheless, the validity of this and other similar tests is controversial when applied to subjects with pain. For instance, it has been reported that more than 40 years ago, individuals with back pain whose origin was not organic, were more likely to present a certain MMPI configuration, the so called "V of conversion" or "neurotic triad", where it was believed that an increase in hypochondria and hysteria scale reflected patients' worries about their own health [45], while a lower score of depression indicated that the patient was indifferent to such worries. Despite the existence of supporting data, other studies would suggest that this specific configuration may represent a sort of adaptation to a chronic disorder and, consequently, it may be a characteristic of patients with chronic health problems, independently of any identified organic etiology [46,47].

To assist caregivers in distinguishing whether a patient's pain had an organic or a psychological origin, Waddel and co-workers [48] suggested the noting of 5 possible signs: 1) pain which is superficial or without anatomic distribution; 2) pain which is induced by any movement which should not be painful but which might appear similar to some other movement which normally would be; 3) pain which seems to vanish when the subject is distracted; 4) focal disturbances, such as hypostenia or sensorial deficit, without anatomic distribution; 5) a patient's hyper-reaction during clinical examination.

Since the accurate evaluation of pain is controversial and possibly influenced by a large number of factors, it might be useful to introduce the concept of "behavior in pathology" [30,49], which could include considerations such as the perception of pain, any decision regarding treatment and the person responsible for this decision, the meaning of pain for the patient and the way the patient talks about the pain and its effect on his/her functioning. The cultural background, the socio-economical conditions, the psychological functioning, the experiences, memory and learning are some of the factors which may influence behavior in pathology. Although the concept of behavior in pathology has been applied more frequently to chronic pain, several observations indicate that some of these factors play a significant role also in acute pain. In a pioneering study, Beecher [50] observed that soldiers who were very severely wounded in battle often did not refer to pain or if they did, it was often to a much lesser extent than might have been expected, given the severity of the wound in question. The author hypothesized that this might reflect the relief of soldiers who had realized that now that they were wounded they would be removed from the field of battle and that their life would no longer be at risk; on the other hand, life stressors might contribute to the development of chronic pain [51].

It has also been demonstrated that a person's knowledge and expectation of potential pain can modify his/her response. Egbert and co-workers [52] found that the pre-operatory psychological preparation of the patient had a significant influence on post-surgical recovery as well as the amount of pain felt. Other studies indicated the existence of significant differences between cultures and ethnic groups with regard to how persons report pain and their response to it. In pathology, the kind of behavior which is generally considered acceptable varies from culture to culture. Zborowski [53] studied experiences of pain amongst "old-Americans" (i.e. of Anglo-Saxon origin), Italo-Americans and American Jews and he observed that the "old-Americans" were generally stronger and had a greater tendency to avoid social contact when experiencing pain, compared with the others. Similarly, Sternbach and Tursky [54] compared different ethnic groups and found that there were differences in the tolerance of pain between the groups and this was subsequently reported also by other authors [55-57].

## Psychiatric comorbidity

Although there is unquestionably some connection between pain and various psychiatric disorders, its exact nature is a matter of debate. The majority of studies on this topic have focused on the frequency of psychiatric disorders amongst patients reporting pain, but a few studies investigating pain in psychiatric subjects have reported this as a common problem. Deplaine and coauthors [58] found that 38% of 227 psychiatric inpatients reported pain; Chaturvedi [59] identified pain symptoms in 18% of patients in a psychiatric department. Both studies underlined that pain was more frequent amongst patients affected by neurosis, as compared with those suffering from schizophrenia or psychotic disorders. Chronic pain would seem to be associated most frequently with a variety of depressive disorders such as major depression, dysthymia and adaptation disorder with depressive mood. The available literature reports a variability in the prevalence of chronic pain ranging from 10 to 100% [60,61]. The heterogeneous results may reflect the difficulty in applying this diagnosis to patients with pain, but may also be related to differences in the evaluated populations. These disorders have been identified in patients with an evident organic etiology for pain as well as in subjects without it, although the disorders would seem to be more common in the latter group [62].

Although some patients have been hypothesized as developing pain as a response to depressive symptoms, other viewpoints have been put forward. According to Engel [63], there may be individuals who, as a result of specific psychological factors, may be considered "prone to pain". Similarly, Blumer and Heilbronn [64] hypothesized that associated mental disorders might precede and/or create a predisposition towards pain and they described an individual prone to pain, for whom it represents a form of masked depression. Some studies indicate that depressive disorders and alcohol dependence are more common in first-degree relatives of patients with chronic pain, thus suggesting a possible environmental or biological predisposition to the development of pain [32,62,65-67]. Other studies would suggest that depression is secondary to pain [31,68], or that both problems can coexist independently or as a result of a common psychological or neurochemical background [62,69,70].

Even some forms of acute pain appear to be associated frequently with other mental disorders; Beithman and co-authors [71] observed that more than 30% of patients with chest pain and normal coronary circulation assessed by means of coronarography met the criteria for panic disorder.

## Depression

The National Health and Nutrition Study underlined that depression was more frequent in older patients, irrespective of the presence of any pain [72]; nevertheless, the relationship between pain and depression is still controversial [73]. Approximately 60% of patients with depression present pain at the moment of the diagnosis [74,75]. The presence of depressive disorder may increase the risk of developing a musculoskeletal pain, headache and chest pain 3 years later on [12,76-78]. Elderly patients with depression are at increased risk for cervical, lumbar and hip pain. Even after 8 years, patients with a previous depression still had a double risk of developing chronic pain, as compared with non-depressed patients.

Depression prevalence was 12% in individuals with three or more pain-related symptoms, as compared with 1% of patients without pain or one only pain-related symptom, amongst 1016 subjects [79]. Major depression prevalence in patients with chronic lumbar pain was three times higher than in the general population [80]. In groups of patients with symptoms with no medical explanation, such as lumbar pain or dizziness, two thirds had a history of recurrent major depression compared with less than 20% in the control group with medical disorders [31,81,82]. In patients with chronic pain included in a program of pain evaluation, between 8% and 50% had suffered from major depression [83]. Where pain led to a loss of independence or motricity with impairment of social functioning, the risk of depression increased significantly [84]. Individuals with chronic problems are at a higher risk of developing lifetime depression and it hasbeen hypothesized that this may be the case with particularly vulnerable individuals.

Depression is not simply a comorbid condition when associated with chronic pain, since it interacts by increasing morbility and mortality. Patients with chronic pain and depression have reported major pain, a reduction in control over life events and a higher probability of coping with stress by adopting passive strategies [74]. They have reported also that this interfered with their lives and they exhibited pain-related behaviors more frequently than did those patients with chronic pain but without depression [85,86]. The presence of pre-operatory depression in patients who underwent lumbar disc surgery was a predictor of a worse result during a 1-year follow-up [87]. In patients with rheumatoid arthritis, depressive symptoms were significantly associated with negative functional results and with a greater utilization of health services [88]. Depression may be a significant predicting factor of pain persistence and of anticipated retirement [12,89]. However, the measurement of pain threshold, by means of different techniques, has led to controversial findings [73].

Patients with headache, chronic abdominal pain and painful orthopaedic syndromes have reported a higher level of suicide thoughts and attempts than those without [90]. In those patients reporting a suicide attempt, 25% suffered from a somatic disease and 21% took pain relievers daily for the pain [91]. Patients with chronic pain are at two to three times greater risk of a completed suicide than the general population [92]. Oncological patients with pain and depression showed a higher likelihood of committing suicide, by searching for information regarding the possibility of suicide or withdrawal from treatment; however, if affected by pain but without depression, they did not request euthanasia or assisted suicide [93], therefore depression must be treated correctly and not simply "understood" as the unavoidable result of chronic pain.

## Anxiety

Patients with different painful syndromes, such as chronic lumbar pain, chronic cervical pain following whiplash or chronic pain due to prostatic cancer, showed an increased risk of anxiety syndromes or disorders [94-97]. At least 50% of patients show anxiety symptoms and 19% have an anxiety disorder such as panic disorder or generalized anxiety disorder [32,98,99].

A prospective study involving 1007 young adults found that a history of headache was associated with a higher risk (odds ratio = 12.8) of panic disorder [100]. On the other hand, anxiety disorders are associated with high somatic preoccupation levels and physical symptoms. In a study of patients with panic disorder, at least 40% described chronic pain symptoms and more than 7% took pain relievers daily [101]. When compared with other patients with panic disorder, this sub-group had a higher frequency of abnormal behaviors related to the disease. The self-evaluation of pain intensity in patients with rheumatoid arthritis was significantly influenced by the presence of anxiety and depression, even when the factor related to the activity of the main disease was controlled [102].

## Substance use

Terms such as addiction, misuse, excessive use and abuse are usually applied inconsistently to describe different behaviors and they may complicate the interpretation of several studies. Frequently the use of drugs is not reported completely and this impairs the accurate evaluation of their use when made by patients with chronic pain [103]; in patients with chronic pain who develop an addiction to new substances, usually this derives from drugs prescribed by the physician [104,105]. The mechanism underlying the relapse of substance abuse amongst these patients is not well-known and it is based upon several factors; however, a period of pain followed by a remission after the use of drugs constitutes a typical example of a situation likely to lead to their use in the future [106]. In a study performed in a group of outpatients consulting a centre specialized in the treatment of pain, almost 90% were taking drugs [107], opioids were prescribed in 70% of the cases, while antidepressants and benzodiazepines were taken by 25% and 18% of the patients, respectively. Amongst this population, 8% met DSM-III-R criteria for substance abuse or addiction; however, misuse and drug abuse were not limited to psychoactive substances. In a review of 24 studies on drug and alcohol dependence in patients with chronic pain, only 7 met the standard criteria for substance abuse disorders, and the prevalence ranged from 3.2% to 18.9% [108]. In a study carried out in patients with chronic lumbar pain, 34% had a substance abuse disorder, which in all cases had already been present before the onset of pain [94]. Individuals with a history of substance abuse ran a major risk of resuming substance abuse while suffering from chronic pain, and also of suffering further physical damage [109].

Research related to substance abuse has revealed abnormalities in the perception and tolerance of pain. In a study carried out in patients with cocaine and opioid abuse, tolerance of the cold pressor test was significantly lower amongst those who were still indulging in substance abuse, than amongst those who were no longer doing so [110]. Alcoholic patients and non-alcoholic males with a high genetic risk of alcoholism, have a higher sensitivity than control subjects to painful stimuli, but this difference in sensitivity was reduced by the intake of pharmacological doses of alcohol [111]. This increase in sensitivity and the relief due to substance abuse would suggest a possible explanation of substance abuse in patients with chronic pain.

## Pharmacological Treatments

This review will focus mainly on pharmacological strategies with special attention being paid to the use of psychotropic compounds. However, apart from pharmacological agents, there exist several heterogeneous techniques for the treatment of pain, such as surgical intervention, nerve blocking, physiotherapy, acupuncture or electrical stimulation, just to mention a few. Among psychotherapies, moreover, cognitive-behavioral therapies (CBT) are considered effective treatments for a variety of pain problems [112-114]. A discussion of these techniques would go beyond the scope of this paper and could be found elsewhere [115].

## Opiates

The use of opiates for non-malignant chronic pain is a matter of debate [116]. For a long time, opiates were reserved for the treatment of painful syndromes related to cancer or to those of acute pain; however, different studies aimed at assessing the risk of abuse have provided reassuring results, in particular that carried out in a group of more than 12000 patients with medical disorders who had been treated with opiates, which showed that, of those without a past history of abuse, only 4 developed drug dependence [117,118]. Open-label and controlled trials support the safety and the efficacy of opiates in the treatment of non-malignant chronic pain.

The most common side-effect following chronic use is the reduction of gastro-intestinal motility, with consequent constipation [119]. However, the main reason why these drugs are not widely prescribed is that there is concern regarding possible cognitive impairment. Such literature as is available has reviewed only some of the effects shown by neuro-psychological tests or electroncephalogram modifications in patients taking combinations of drugs, mainly sedatives and hypnotics [120-123]. When the opiate treatment is not effective, the drug should be suspended gradually in order to decrease withdrawal symptoms. The opiate withdrawal is usually dangerous only in those patients at risk with regard to an increase in the sympathetic tone, that is, for instance, in those who show an increase in intracranial pressure or unstable angina. In any event, however, it should be borne in mind that opiate reduction usually leads to a return of the original painful symptoms (rebound pain).

## Antidepressants

After the first study reporting the effectiveness of imipramine in the treatment of trigeminal neuralgia [124], antidepressants, especially tricyclics (TCAs), have frequently been prescribed for the treatment of several chronic painful syndromes, but mainly for neuropathic pain. Various different studies have suggested that the antidepressant effects on pain are mediated by the blockade of the norepinephrine and serotonin re-uptake which increases neurotransmitter levels and, therefore, potentiates the activation of the descendent inhibitory pathways [62,125]. Other aspects of monoaminergic systems are involved in the analgesic action of antidepressants, for example, β-adrenergic receptors may mediate the analgesic effects of desipramine and nortriptyline [126]. In general, amitriptyline, or TCAs with a similar pharmacological profile, are considered the most effective drugs; however, randomized controlled clinical trials have not demonstrated the existence of any significant differences between TCAs [127]. In a review of 39 controlled studies versus placebo, in 80% of the cases, antidepressants proved superior to the placebo [128]. TCAs have been the most effective drugs for pain remission in neuropathic pain and headache, and the analgesic activity would seem to be independent of any effect on mood [129-134]. A recent controlled randomized study of nortriptyline versus placebo in patients with chronic lumbar pain without depression, showed a significant reduction in the scores related to pain intensity [135]. The results of studies which have assessed the plasma levels of drugs necessary for pain remission are still controversial and clear guidelines have not been established as yet [136]. Nortriptyline, the main amitriptyline metabolite, induces less sedation and less orthostatic hypotension than imipramine, but has an analgesic potency comparable to that of amitriptyline [137,138]. The cost of pain treatment with TCAs is usually much lower than that with other antidepressants or drugs with an analgesic action, such as β-blockers or calcium-antagonists [139]. Selective serotonin re-uptake inhibitors (SSRIs) have a weak antinociceptive action in animal models of acute pain [140-142]. Several studies have investigated the role of different serotonin receptor subtypes in the mechanisms of pain, but they are very controversial [143-145]. SSRIs do not seem to be as effective as TCAs for chronic pain; for instance, desipramine is superior to fluoxetine in the treatment of painful peripheral diabetic neuropathia [146]. Recently these drugs have been shown to be effective and well-tolerated in the treatment of headache, in particular of migraine [147,148]. The role of biogenic amine in the descendent inhibition of pain would suggest the potential efficacy of all antidepressants, whatever their mechanism of action [149]. In studies of thermic nociception, the norepinephrine re-uptake inhibitors, such as reboxetine, and the dopamine re-uptake inhibitors, such as bupropion, may produce an antinociceptive effect [150]. Trazodone has been effective in reducing pain in a double-blind controlled study carried out in patients with chronic lumbar pain [151,152]. In an animal model of neuropathic pain, venlafaxine, a compound inhibiting both the re-uptake of norepinephrine and serotonin (SNRI), was shown to prevent the development of hyperalgesia [152]. Of note, duloxetine, a balanced SNRI, has been recently considered a new treatment option for patients with neuropathic pain after showing promising results in controlled trials [153].

## Anticonvulsants

Anticonvulsants are considered the most effective drugs in the treatment of trigeminal neuralgia, of diabetic neuropathy, and of migraine recurrence [137]. Phentoine was the first drug found to be effective in the treatment of trigeminal neuralgia in 1942 [154]. Carbamazepine is the most studied anticonvulsant which has proven to be effective in the treatment of neuropathic pain [155]. However, anticonvulsants have different pharmacological actions which may have a role in analgesia with potentially useful connections with a series of chronic pain syndromes. Several anticonvulsants block the activity of sodium channels and, therefore, would stabilize the pre-synaptic neuronal membrane, decrease the release of excitatory neurotransmitters and reduce the spontaneous firing of damaged or recovering nociceptive fibres. Phentoine regulates many aspects of nervous functioning, such as the activity of ATPase, synaptic transmission, the release of neurotransmitters and ion conduction [156]. Carbamazepine reduces the tryptophan concentration binding plasma proteins and consequently increases serotonin levels in rat brain [157]. The use of carbamazepine, however, is restricted by unwanted side-effects such as sedation, ataxia and plastic anemia. Therapeutic plasma levels have not been defined clearly, but there is evidence to suggest that low levels may be effective in reducing pain [158].

New classes of anticonvulsants have different pharmacological actions which may prove to be analgesic. Valproic acid is more frequently used in migraine prophylaxis but it is effective also in the treatment of neuropathic pain [159]. In a study on migraine, valproate proved to be effective in the prophylactic treatment of more than two thirds of the patients, with minimal side-effects such as nausea, dizziness or tremors [160]. The mechanism of action of valproate is probably related to an increase in GABA levels, mediated by the inhibition of GABA transaminase and the increase of GABA synthesis. Valproate is usually well-tolerated, but it requires regular monitoring because of its potential hepatotoxicity and suppression of bone marrow.

In open-label studies, gabapentin has been shown to reduce pain in multiple sclerosis, migraine, post-herpetic neuralgia (PHN) and reflex sympathetic distrophy [161,162]. The mechanism of action of gabapentin may be related to the binding to the α_2_δ subunit of the voltage-dependent calcium channels [163]. Clinical cases exist which suggest that lamotrigine may be effective in reducing phantom limb pain, post-stroke central pain and PHN [164,165]. The mechanism of action of lamotrigine may result from the reduction in the long-term excitatory effects of glutamatergic nociceptive transmission mediated by NMDA receptors, but it also blocks the voltage-dependent sodium channels [166-168].

## Benzodiazepines

Benzodiazepines are frequently prescribed for insomnia and anxiety in patients with chronic pain [169,170]. In a review of published literature, only a few conditions involving chronic pain, such as trigeminal neuralgia, tensive headache and temporal-mandible joint disorder, seem to improve after treatment with benzodiazepines [171]. It is difficult to assess the positive effects of benzodiazepines, however the negative effects have been studied thoroughly and they amount to more than the usual worries related to abuse, dependence, abstinence or secondary effects on mood. Elderly subjects are particularly sensitive to the side-effects of benzodiazepines, such as sedation [172-176]. Benzodiazepines determine also a cognitive impairment, as demonstrated by the abnormalities of neuropsychological tests and electroencephalograms [120].

## Conclusion

Pain is a common problem in medical practice and it continues to be underestimated and undertreated despite the existence of effective treatments.

The psychological aspects of pain are evident; nevertheless these are rarely assessed or managed as they should be. Furthermore, pain can often represent a non-specific sign or symptom of specific psychiatric disorders, such as depression or anxiety, or it can constitute a disorder in itself, as acknowledged by current psychiatric classification systems and as a result, in most cases, psychiatric intervention can prove beneficial.
